# Molecular Evidence of *Bartonella* Species in Ixodid Ticks and Domestic Animals in Palestine

**DOI:** 10.3389/fmicb.2016.01217

**Published:** 2016-08-04

**Authors:** Suheir Ereqat, Abdelmajeed Nasereddin, Muriel Vayssier-Taussat, Ahmad Abdelkader, Amer Al-Jawabreh, Taher Zaid, Kifaya Azmi, Ziad Abdeen

**Affiliations:** ^1^Biochemistry and Molecular Biology Department, Faculty of Medicine, Al-Quds University, Abu DeisPalestine; ^2^Al-Quds Nutrition and Health Research Institute – Faculty of Medicine, Al-Quds University and Al-Quds Public Health Society, Abu DeisPalestine; ^3^L’Unité Mixte de Recherche (UMR), Biologie Moléculaire et Immunologie Parasitaires (BIPAR), Institut National de la Recherche Agronomique (INRA)–École Nationale Vétérinaire d’Alfort (ENVA)–Agence Nationale de Sécurité Sanitaire de l’Alimentation, de l’Environnement et du Travail (ANSES), ParisFrance

**Keywords:** bartonellosis, *B. chomelii*, *B. rochalimae*, *B. bovis*, *Rhipicephalus*, *Hyalomma*, Palestine

## Abstract

Ticks play an important role in disease transmission as vectors for human and animal pathogens, including the Gram-negative pathogen *Bartonella*. Here, we evaluated the presence of *Bartonella* in ixodid ticks and domestic animals from Palestine. We tested 633 partly engorged ticks and 139 blood samples from domestic animals (dogs, sheep and camels) for *Bartonella* using ITS-PCR. *Bartonella* DNA was detected in 3.9% of the tested ticks. None of the ticks collected from sheep and goats were positive for *Bartonella*. Seventeen *R. sanguineus* ticks (17/391; 4.3%) collected from dogs were infected with *B. rochalimae* (*n* = 10), *B. chomelii* (*n* = 6), and *B. koehlerae* (*n* = 1). Four *H. dromedarri* ticks (4/63; 6.3%) obtained from camels were infected with *B. bovis* (*n* = 2) and *B. rochalimae* (*n* = 2). Among canine blood samples (*n* = 110), we found one asymptomatic female dog to be infected with *B. rochalimae* (0.9%). The detection of zoonotic *Bartonella* species in this study should raise awareness of these vector-borne diseases among physicians, veterinarians and public health workers and highlight the importance of surveillance and preventive measures in the region.

## Introduction

Tick-borne diseases comprise a group of globally distributed and rapidly spreading illnesses caused by a range of pathogens. Molecular approaches make it possible to screen ticks for pathogens of veterinary and public health importance and perform detailed epidemiological studies (Sparagano et al., 1999). Bartonellosis is an infectious disease caused by bacteria from the genus *Bartonella*, which infect erythrocytes and endothelial cells in humans (Harms and Dehio, 2012). These pathogens are transmitted by biting arthropod vectors and infect a wide range of wild and domestic mammals, including rodents, cats, dogs, and cattle. In humans, *Bartonella* is responsible for emerging and reemerging diseases worldwide with presentations that range from subclinical or self-limiting infection to severe, life-threatening disease.

Different *Bartonella* species appear to be adapted to specific mammalian hosts (Breitschwerdt and Kordick, 2000; Vayssier-Taussat et al., 2010). For example, cats are the main reservoir for *B. henselae*, which causes cat-scratch disease. Cats are infected through the bites of cat fleas, while humans are directly infected through scratches or bites from an infected cat (Billeter et al., 2008).

By contrast, diverse species of *Bartonella* infect ruminants. *B. bovis* is the most commonly reported species in cattle, where it is associated with bovine endocarditis (Maillard et al., 2004; Erol et al., 2013). This species has been described in beef and dairy cattle worldwide, including North and South America, Italy, France, Israel, Taiwan and peninsular Malaysia (Martini et al., 2008; Cherry et al., 2009; Saisongkorh et al., 2009; Tsai et al., 2011; Bai et al., 2013; Rudoler et al., 2014; Kho et al., 2015). Another species, *B. chomelii*, has been isolated from cattle in France and Spain (Maillard et al., 2004; Antequera-Gomez et al., 2015). *B. rochalimae* infects humans, domestic animals and wild carnivores (Schaefer et al., 2012), so there has been much interest in its zoonotic potential (Eremeeva et al., 2007; Chomel et al., 2009).

Although transmission of *Bartonella* to humans typically occurs through traumatic contact with infected animals or by blood-sucking insect vectors such as fleas, lice and sand flies, this group of species has also been widely reported from ticks. The potential for involvement of ticks in transmission of *Bartonella* spp. has been suggested by several molecular and serological epidemiological studies (Billeter et al., 2008). *Bartonella* was first detected in questing ticks from the USA, including *Ixodes pacificus, Dermacentor*, and *R. sanguineus* (Chang et al., 2000). Additional surveys conducted in the Netherlands, France, Poland, and Austria have demonstrated the presence of *Bartonella* DNA in *Ixodes ricinus* ticks obtained from vegetation (Billeter et al., 2008). In France, the species *B. henselae* has been identified in *Ixodes ricinus* isolated from vegetation (Vayssier-Taussat et al., 2013), as well as from humans exposed to tick bites (Vayssier-Taussat et al., 2016).

Clinical studies have supported transmission of *Bartonella* by ticks to humans, as infections have occurred after tick bites without any known contact with other arthropods (Morozova et al., 2005; Billeter et al., 2008). *Bartonella* infection was reported in three patients with scalp eschar and neck lymphadenopathy following tick bites (Angelakis et al., 2010). A recent study by Vayssier-Taussat et al. (2016) identified potentially zoonotic *Bartonella* strains in symptomatic patients who reported tick bites. Natural co-infections with *Bartonella* species and other tick borne pathogens such as *Babesia*, *Anaplasma*, and *Borrelia* have also been demonstrated (Hofmeister et al., 1998; Angelakis et al., 2010). In particular, co-infections of *Bartonella* and *Borrelia* have been reported in humans from the USA and Europe (Billeter et al., 2008). These observations provide indirect evidence for tick-borne transmission, even in the absence of direct proof of tick vector competence for *Bartonella* (Cotte et al., 2008; Reis et al., 2011). Furthermore, experimental transmission studies using infected ticks and live susceptible animals support the role of ticks in the natural lifecycles of some *Bartonella* species (Cotte et al., 2008; Reis et al., 2011).

In Palestine, *Bartonella* DNA has been detected in 22% (64/289) of fleas collected from various animal hosts (dogs, cats and rodents) (Nasereddin et al., 2014). Several *Bartonella* species have been identified in Palestinian samples, including *B*. *clarridgeiae, B. henselae, B. koehlerae, B. tribocorum, B. elizabethae*, and *B. rochalimae* (Nasereddin et al., 2014).

In our previous studies, when we screened ticks from domestic animals, we identified several tick-borne pathogens, including *Rickettsia* from the spotted fever group, *Babesia* and *Hepatozoon* (Ereqat et al., 2016; Azmi et al., 2016). Given the potential role of ticks as a source of zoonotic *Bartonella* infection in humans (Vayssier-Taussat et al., 2016), we wished to determine whether ticks carry *Bartonella* in Palestine. More specifically, we set out to extend our previous surveys and to assess the presence of *Bartonella* in a set of previously studied ticks and in the blood samples collected from their animal hosts throughout Palestine.

## Materials and Methods

### Study Sites, Tick, and Animal Samples

**FIGURE 1:**
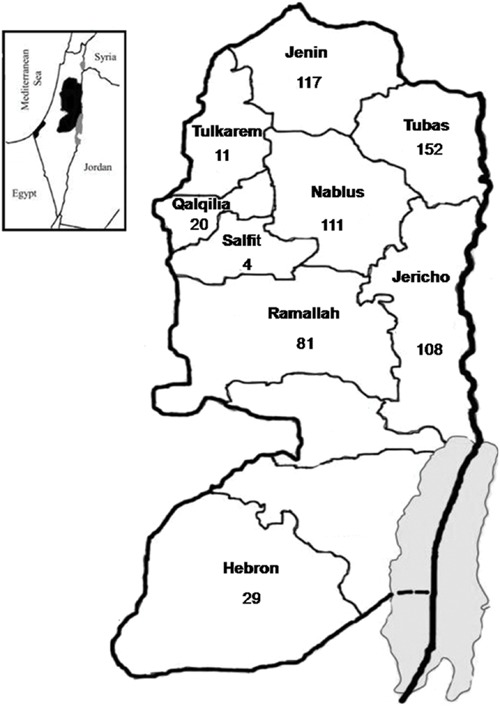
**Distribution of Ixodid ticks collected from nine districts in Palestine from which *Bartonella* DNA was detected**.

A total of 633 hard ticks were collected during January to October 2014. The ticks were collected from dogs, sheep, goats, and camels in nine districts of Palestine (Hebron, Jenin, Jericho, Nablus, Qalqilia, Ramallah, Salfit, Tubas, and Tulkarem), located in three zones in the central, northern and southern regions of the country (**Figure 1**). Ticks were identified based on morphological characteristics (Feldman-Musham, 1954).

A total of 110 blood samples were collected from outdoor domestic dogs, with simultaneous tick collection from the locations mentioned above (17 from Hebron, 25 from Jenin, 18 from Jericho, three from Nablus, 21 from Ramallah, 13 from Salfit, and 13 from Tulkarem). All samples were collected in EDTA-anticoagulant tubes and stored at -20°C until further use. Study animals were selected irrespective of sex and age. In addition, 29 blood samples were obtained from camels (*n* = 19), sheep (*n* = 7), and goats (*n* = 3) from Jenin, Nablus, and Jericho, in January 2016. None of the animals showed clinical signs; all were apparently healthy at the time of sampling. The animal owners were verbally informed about the goals of this research and the sampling protocol. All owners gave their written informed consent to collect blood from their animals. The ethics committee at Al-Quds University approved the study.

### DNA Extraction

DNA was extracted from each tick using a DNA extraction kit (QIAGEN GmbH, 40724 Hilden, Germany) following the manufacturer’s instructions. The eluted DNA (100 μl) was stored at -20°C until used as templates for PCR amplifications. DNA was extracted from whole blood (200 μl) following the QIAamp animal blood and Tissue Kit procedure (QIAGEN GmbH, Hilden, Germany), adjusted in 200 μl of Tris- EDTA (TE) buffer and stored at -20°C until further use.

### Molecular Detection and Identification of *Bartonella* Species

For screening, conventional PCR was performed on all tick and blood samples (*n* = 772) targeting the Intergenic Transcribed Spacer (ITS) locus, using the following forward and reverse primers: (321s: 5′AGATGATGATCCCAAGCCTTCTGG and H493as: 5′-TGAACCTCCGACCTCACGCTTATC) as previously described (Maggi and Breitschwerdt, 2005; Gutierrez et al., 2014). PCR reactions were performed in 25-μl Syntezza PCR ready mix (Syntezza, Jerusalem), containing 1 μM of each set of primers and 5 μl of the extracted DNA. The thermal cycling procedure was as described previously (Norman et al., 1995; Renesto et al., 2001; Maggi and Breitschwerdt, 2005). Samples of PCR grade water were included as a negative (no-DNA) controls. To confirm amplicon identity, all PCR products from the positive samples underwent DNA sequencing; the nucleotide sequences were compared to those present in GenBank database using the Basic Alignment Search Tool (BLAST).^1^ Statistical analysis was done using the SPSS program v20.

## Results

### *Bartonella* DNA in Animal Blood

Among the canine blood samples (*n* = 110; 22 female: 88 males), one female dog sample was positive for *Bartonella*, yielding a PCR-amplified ITS sequence that showed 99% sequence identity to the reference sequence of *B. rochalimae* (FN645466.1). None of the blood samples from camels (*n* = 19), sheep (*n* = 7), and goats (*n* = 3) were positive for *Bartonella*.

### *Bartonella* DNA in Ticks

A total of 633 hard ticks (292 female, 286 male, and 55 Nymph ticks) were obtained from 188 animals (137 dogs, 38 sheep, 10 camels, and three goats), residing in nine districts throughout Palestine. The geographic distribution of collected ticks was shown in **Figure 1**. All ticks representing three genera and seven species [*Haemaphysalis parva (n = 43)*, *Haemaphysalis adleri (n = 13)*, *Rhipicephalus turanicus (n = 91), Rhipicephalus sanguineus (n = 391), Rhipicephalus bursa (n = 7), Rhipicephalus* sp.(*n = 20), Hyalomma dromedarii (n = 63)*, and *Hyalomma impeltatum* (*n* = 5)] were screened for *Bartonella* DNA.

Overall, 25 ticks (3.9%; 9 females, 11 males, and 5 nymphs) were positive for *Bartonella* DNA by ITS-PCR. Of these 21 ticks were collected from dogs and four from camels. None of the ticks from sheep or goats were positive for *Bartonella* (**Table 1**). Identification of *Bartonella* was successful for 21 positive samples (84%). The initial attempt to sequence four amplicons failed and no additional DNA samples were available. Seventeen *R. sanguineus* ticks were infected with *B. rochalimae* (*n* = 10), *B. chomelii* (*n* = 6), and *B. koehlerae* (*n* = 1). All these ticks were obtained from dogs.

**Table 1:** The overall prevalence of *Bartonella* DNA in ticks species and their associated animal hosts.

Ectoparasites	No.	pos. PCR	Infection rate	Animal host
*Rhipicephalus turanicus*	91	0	0	Dog, sheep
*Rhipicephalus sanguineus*	391	20	5.1	Dog, sheep, Goat,
*Rhipicephalus bursa*	7	0	0	Sheep
*Rhipicephalus* spp.	20	1	5	Dog
*Haemaphysalis adleri*	13	0	0	Dog, sheep
*Haemaphysalis parva*	43	0	0	Dog, sheep
*Hyalomma dromedarii*	63	4	6.3	Camel
*Hyalomma impeltatum*	5	0	0	Camel
*Total ticks*	633	25	3.9	

Four *H. dromedarii* ticks obtained from a single camel in Hebron and from two camels in Jericho were found to be infected with *B. bovis* (*n* = 2) and *B. rochalimae* (*n* = 2), respectively (**Table 2**). *Bartonella* species identification was based on the highest scoring BLAST hit on GenBank. The comparison of the PCR-amplified ITS sequences from the positive tick samples showed 97–99% sequence identity and 100% coverage when aligned against the reference sequences of *B. bovis* (KR733201.1), *B. chomelii* (KM215714.1), *B. rochalimae* (FN645466.1), and *B. koehlerae* (AF312490.1). Representative partial sequences of the 16S–23S ribosomal RNA intergenic spacer identified in the present study were deposited in GenBank under the following accession numbers: two *B. rochalimae* from a tick and its host (dog) (KX420619, KX420620), *B. chomelii* (KX420617), *B. bovis* (KX420618), and *B. koehlerae* (KX420616).

**Table 2:** Molecular identification of *Bartonella* spp. collected from Ixodid ticks from different localities throughout Palestine.

*Bartonella* spp.	Ectoparasites (*n*)	Site of collection
*B. chomelii*	*Rhipicephalus sanguineus (6)*	Hebron, Tubas
*B. rochalimae*	*Rhipicephalus sanguineus (10), Hyalomma dromedarii (2)*	Nablus, Jenin, Tubas, Ramallah, Jericho
*B. bovis*	*Hyalomma dromedarii (2)*	Hebron
*B. koehlerae*	*Rhipicephalus sanguineus (1)*	Jenin

## Discussion

Here, we report *Bartonella* DNA in ixodid ticks and blood samples from domestic animals from Palestine. The overall prevalence of *Bartonella* DNA in ticks (3.9%) was in agreement with previous screening undertaken worldwide including Czech Republic, United States, Italy and Thailand (Sanogo et al., 2003; Hercik et al., 2007; Billeter et al., 2008, 2012). Several *Bartonella* species have been identified in humans, animals, and their flea vectors in neighboring countries. However, *Bartonella* DNA was not detected in any of the Ixodid ticks examined in Israel and Egypt (Loftis et al., 2006; Harrus et al., 2011).

In our study, four *Bartonella* species were identified: *B. rochalimae*, *B. chomelii*, *B. Bovis*, and *B. koehlerae*. *B. rochalimae was* the predominant *s*pecies among *Bartonella*-positive ticks (12 out of 25 samples; 48%). We examined seven hard tick species for *Bartonella* and found evidence of the pathogen in two of them: the brown dog tick, *Rhipicephalus sanguineus* and the camel tick, *Hyalomma dromedarii*. These findings represent the first detection of *Bartonella* in Ixodid ticks from the Middle East. Cases of human parasitism by the brown dog tick—the most widespread tick in the world—are well documented (Fernandez-Soto et al., 2006). Together, the exposure of animals to arthropod vectors and the proximity of infected animals to humans make some *Bartonella* species potential zoonotic agents.

People dealing with dogs (e.g., veterinarians, shepherds, dog owners and pet shop workers) appear to be at particular risk of exposure to *R. sanguineus* and its pathogens. We found *Bartonella* DNA in a single sample of dog blood, with high sequence identity to a reference sequence from *B. rochalimae* (99% DNA sequence identity; GenBank accession number FN645466.1). The dog that provided the sample was apparently healthy, showing no signs of bartonellosis. Detection of *Bartonella* in asymptomatic dogs has also been reported from Peru (Diniz et al., 2009). We identified *B. rochalimae* in two *R. sanguineus* ticks obtained from the same dog; both tick samples yielded sequences 100% identical to each other and to the sequence obtained from dog. Although the blood and tick samples were collected from dogs at the same time, *Bartonella* DNA was more prevalent in the dog ticks (5.4%) than in the dog blood (0.9%) suggesting that *Bartonella* be carried in partially engorged adult ticks. These findings provide highly suggestive molecular evidence that *R. sanguineus* ticks can act as vectors of animal-associated *Bartonella* infection in Palestine. Furthermore, although the role of dogs as source of human *Bartonella* infection remains unclear, we speculate that they may present a risk for zoonotic transmission similar to that seen with *B. henselae* in cat scratch disease (Chomel et al., 2006).

Recently, a study conducted in Israel confirmed the presence of a novel species of *Bartonella* in camelids, which has been named *Bartonella dromedarii* sp. nov (Rasis et al., 2014). In our study, we identified two species of *Bartonella* (*B. rochalimae* and *B. bovis*) in ticks obtained from camels, although we did not detect *Bartonella* DNA in blood samples from these camels. However, we cannot rule out bloodstream infections in the camels as the source of *Bartonella* in the ticks, as the blood samples were not taken at the same time as the ticks. Furthermore, even if camels are not reservoir hosts for these *Bartonella* species, they may have an important role as mechanical dispersers of infected ticks.

Our discovery of *Bartonella* in camel ticks from the species *Hyalomma dromedarii* is worrying because other species of hard ticks from the genus *Hyalomma* clearly bite humans (Psaroulaki et al., 2005; Bursali et al., 2011) and there are suggestions that *H. dromedarii* can do so too^2^. Other lines of evidence support the role of ticks in the natural cycles of some *Bartonella* species including those pathogenic for humans (Cotte et al., 2008; Reis et al., 2011; Liu and Bonnet, 2014). In particular, ticks can be infected in the larval or nymph stages by ingesting blood from an intermediate host carrying *Bartonella*. The pathogen can then survive in the midgut of ticks during molting and can be transmitted through feeding to an uninfected host.

In the present study, brown dog ticks were found to be infected with *B. chomelii*—a pathogen first isolated from French domestic cattle (Maillard et al., 2004) and found to be the most frequent *Bartonella* species infecting cattle grazing in Spain pastures (Antequera-Gomez et al., 2015). We also describe the first detection of *B. koehlerae* in a *R. sanguineus* tick obtained from dog. This species was first isolated from the blood of two pet cats in California (Droz et al., 1999). Since then, it has been detected in cats and their fleas in France, Israel and Palestine (Rolain et al., 2003; Gutierrez et al., 2013; Nasereddin et al., 2014), has been reported as causing endocarditis in humans and Boxer dogs in Israel (Avidor et al., 2004; Ohad et al., 2010). However, the presence of a microbial agent within a tick does not imply that the tick is a biological vector and might transmit it during the course of blood feeding (Telford and Wormser, 2010).

One of *Bartonella* -positive ticks we obtained from a dog harbored *Rickettsia*, a pathogen that is known to be tick-transmitted (Ereqat et al., 2016). This fits in with evidence from across the world that *Bartonella* is often found in ticks alongside well-known tick-transmitted organisms such as *Anaplasma, Borrelia*, *and Rickettsia* (Angelakis et al., 2010). Other studies supporting the hypothesis that *Bartonella* can be transmitted by ticks include a US report that dogs infected with *Bartonella* were also seropositive for *Anaplasma phagocytophilum* (MacDonald et al., 2004) and a human case study showing that patients infected with *Borrelia burgdorferi* after tick bites also carried *Bartonella* DNA in their blood (Podsiadly et al., 2003).

## Conclusion

The detection of zoonotic *Bartonella* species in this study should increase the awareness of these vector-borne diseases among physicians, veterinarians and public health workers and highlight the importance of surveillance and preventive measures in the region. Additional epidemiologic surveys are required to enhance our understanding of the transmission dynamics of *Bartonella* in Palestine and in other parts of the Middle East.

## Author Contributions

Conceived and designed the experiments: SE, AN, and ZA. Performed the experiments: SE, AA, and TZ. Analyzed the data: SE and AA-J. Wrote the first draft of the manuscript: SE. Directed, revised, and contributed to the writing of the manuscript: AN and V-TM. Final revision and approval of the manuscript to be published: SE, AN, V-TM, AA, AA-J, TZ, KA, and ZA.

## Conflict of Interest Statement

The authors declare that the research was conducted in the absence of any commercial or financial relationships that could be construed as a potential conflict of interest.

## Footnotes

1 http://www.ncbi.nlm.nih.gov/BLAST

2 http://www.ncbi.nlm.nih.gov/pubmed/22662597
